# Effects of cytochalasin D on relaxation process of skinned taenia cecum and carotid artery from guinea pig

**DOI:** 10.1186/s12576-024-00918-3

**Published:** 2024-04-10

**Authors:** Satoko Mihashi, Masaru Watanabe

**Affiliations:** Department of Frontier Health Sciences, Graduate School of Human Health Sciences, Tokyo Metropolitan University, 7-2-10 Higashiogu, Arakawa-Ku, Tokyo, 116-8551 Japan

**Keywords:** Cytochalasin D, Actin, Smooth muscle, Skinned preparation, Relaxation

## Abstract

Actin linked regulatory mechanisms are known to contribute contraction/relaxation in smooth muscle. In order to clarify whether modulation of polymerization/depolymerization of actin filaments affects relaxation process, we examined the effects of cytochalasin D on relaxation process by Ca^2+^ removal after Ca^2+^-induced contraction of β-escin skinned (cell membrane permeabilized) taenia cecum and carotid artery preparations from guinea pigs. Cytochalasin D, an inhibitor of actin polymerization, significantly suppressed the force during relaxation both in skinned taenia cecum and carotid artery. The data fitting analysis of the relaxation processes indicates that cytochalasin D accelerates slow (latch-like) bridge dissociation. Cytochalasin D seems to directly disrupts actin filament organization or its length, resulting in modulation of actin filament structure that prevents myosin binding.

**Supplementary Information:**

The online version contains supplementary material available at 10.1186/s12576-024-00918-3.

## Background

Regulatory mechanisms of contractility in vertebrate smooth muscle are thought to be different from that in striated muscle. In smooth muscle, the contractile force develops dependent on the phosphorylation level of myosin regulatory light chain (MLC), so called “myosin phosphorylation theory” [1, 2]. On the other hand, several intracellular proteins in smooth muscle, such as caldesmon, calponin, myosin light chain and heat shock protein 20 are known to bind actin-tropomyosin [2–7]. In fact, we found that synthetic peptides originated from actin binding region of Troponin I and heat shock protein 20 modulated cell membrane permeabilized (skinned) smooth muscle contraction/relaxation cycle [8–10]. In addition to actin-tropomyosin linked contractile regulation, dynamic changes in length of thin filaments through actin polymerization and depolymerization in living smooth muscle also regulate smooth muscle contractility [11, 12], and previous studies showed that inhibition of actin polymerization suppressed smooth muscle contraction [12, 13]. However, effects of actin polymerization and depolymerization on relaxation process in smooth muscle were not investigated in detail.

Previously, we found that, blebbistatin, a potent inhibitor of myosin II, accelerated skinned smooth muscle relaxation process after Ca^2+^ removal and the results suggest that blebbistatin accelerates slow (latch-like) bridge dissociation, since the slow force decay during relaxation by Ca^2+^ removal in skinned smooth muscle is thought to reflect the “latch”, originally reported and hypothesized by Dillon et al. [14], or the “latch-like” state of smooth muscle [2, 9, 10, 15–17] (see “Discussion” in detail). The experimental evidence noted above strongly suggests that direct inhibition of actin-myosin interaction accelerates the relaxation process by the Ca^2+^ removal in smooth muscle through inhibition of “latch-like” slow cycling bridge. Therefore, in the present study, we aimed to investigate whether modulation of polymerization/depolymerization of actin filaments affects relaxation process in smooth muscle in detail.

To modulate actin polymerization level experimentally, cytochalasin D (CC-D), an inhibitor of actin polymerization that act specifically on actin, was used in this study. CC-D is a low molecular weight compound, permeated cell membrane isolated from fungal metabolites, has a high affinity for the barbed ends of actin filaments (the dissociation constant for binding; Kd 2 nM) and inhibits actin polymerization [18]. CC-D is known to inhibit intact smooth muscle contraction irrespective of MLC phosphorylation level [13].

## Methods

All animal experiments were approved by Tokyo Metropolitan University (A2-3, A3-19) and performed at Tokyo Metropolitan University at Arakawa, and the animal experimental procedures conformed to the “Guidelines for Proper Conduct of Animal Experiments” approved by the Science Council of Japan. The taenia cecum and the carotid artery were removed from male Hartray Guinea pig (c, 250 g) after euthanasia of the animals under deep anesthesia with pentobarbital (Somnopentyl, Kyoritsu Seiyaku Co., Tokyo, Japan), then kept in a normal extracellular solution (NES; described below).

A small strip of taenia cecum or carotid artery (1.5–3.0 mm long and 0.1–0.2 mm wide) was attached to a force measuring apparatus which was connected to a force transducer (ULA-10GR, Minebea Mitsumi Inc., Kanagawa, Japan), then skinned with β-escin at 200 μM and A23187 at 10 μM in a Relaxing solution (with ATP; see Table 1) to destroy cell membrane and sarcoplasmic reticulum, respectively. After skinning, the preparation was passively stretched to a steady level (resting tension, up to 10 μN). The measured isometric force was recorded using a software LabChart7 (ADInstruments JAPAN, Nagoya, Japan) [19, 20].

**Table 1:** Major component of the artificial intracellular solutions (mM)

	Ma^2+^	Ca^2+^	Nucleotide	Creatin phosphate
Relaxing solution with ATP	1.0	0	MgATP 1.0	20
Relaxing solution with CTP	1.0	0	MgCTP 1.0	20
Rigor solution	1.0	0	0	0
Ca^2+^-activation solution	1.0	0.01	MgATP 1.0	20

## Experimental procedure

Application of Ca^2+^ at 10^–5.0^ M with calmodulin at 10^–6.0^ M to the skinned preparation induced the maximal Ca^2+^-induced contraction. When the contractile force reached a steady level, the preparation was immersed in Relaxing solution (with cytidine triphosphate; CTP) in the presence or absence of CC-D to elicit relaxation process by quick removal of Ca^2+^ from intracellular space. To avoid any effects of CC-D on phosphorylation process [13], the Relaxing solution (with CTP) contained MgCTP instead of MgATP, since CTP is a substrate for myosin ATPase, but not for any other kinases [21, 22]. In some preparations, the relaxation process was measured in the absence of any nucleoside triphosphate using Rigor solution (without ATP or CTP) or Relaxing solution (with ATP). In the control experiments, 1% of dimethylsulfoxide (DMSO, Sigma) was added in Relaxing solution (with CTP), Rigor solution (without ATP or CTP), or Relaxing solution (with ATP) as a vehicle control. Since CC-D effects on contraction was similar irrespective of CC-D pretreatment [13], and application of CC-D during Ca^2+^ activated contraction before the relaxation process changes the force level at the begening of the relaxation process, we did not pretreated CC-D before Ca^2+^ removal.

### Solutions and chemicals

NES contained (in mM); 150 NaCl, 4 KCl, 2 CaCl_2_, 1 MgCl_2_, 10 glucose, 10 2-[4-(2-hydroxyethyl)-1-piperazinyl]ethanesulphonic acid (Nacalai Tesque, Kyoto, Japan), and 50 μU/ml insulin (Sigma), and pH was adjusted with Tris(hydroxymethyl) aminomethane (Tris; Nacalai Tesque)/H_2_O to pH 7.40 at 30 ℃. Artificial intracellular solutions for skinned preparations contained (in mM); 0.85 Mg (methanesulfonate)_2_, 1 MgATP (1.35 total ATP Na_2_, Roche Diagnostics, Mannheim, Germany) or 1 MgCTP (1.35 total CTP-Na, Sigma), 20 creatine phosphate Na_2_ (CrP; Nacalai Tesque), 10 etylene glycole-bis (2-aminoetyl) tetraacetic acid (EGTA; Nacalai Tesque). K (methanesulfonate) (Nacalai Tesque) was added to the solutions to keep the ionic strength at 200 mM, and pH was adjusted with 20 mM 1,4-piperazinediethanesulophonic acid (PIPES; Nacalai Tesque) and KOH (FUJIFILM Wako Pure Chemical Corporation, Osaka, Japan) to 7.0 at 30 ℃, which were prepared according to the method of Horiuti [20]. Relaxing solution (with CTP) contained MgCTP instead of MgATP. Ca^2+^-activating solution (at 10^–5.0^ M) were prepared by mixing the Ca^2+^-EGTA solution containing 10 mM EGTA and 9.64 mM Ca (methanesulfonate)_2_ with 1 μM calmodulin (FUJIFILM Wako Pure Chemical Corporation) with the artificial intracellular solution. The apparent dissociation constant of Ca^2+^-EGTA was assumed to be 10^–6.4^ M. Rigor solution (without ATP or CTP) contained (mM); 0.85 Mg (methanesulfonate)_2_, 10 EGTA. K (methanesulfonate) was added to the solutions to keep the ionic strength at 200 mM, and pH was adjusted with 20 mM PIPES and KOH to 7.0 at 30 ℃. CC-D (Sigma) was dissolved into 1% DMSO. Major components of the artificial intracellular solutions were noted in Table 1.

### Data analysis of the mechanical properties

The measured isometric tension level was normalized as relative tension as below;

$$ {\text{Relative tension}} = \,\left( {\text{an observed tension{-}the  resting tension}} \right)/\left( {{\text{maximal tension of the Ca}}}^{{2 + }} {\text{- induced contraction{-}the resting tension}} \right). $$

The relaxation process induced by Ca^2+^-removal of the skinned preparations was kinetically analyzed by data fitting to an equation originally hypothesized by Mihashi et al. [17] as below;

1 $$ {\text{Force}} \, \left( {\text t} \right){\text{ = Force}}\;(0) \times \left[ {e^{{( - {\text t}/}{\tau\,{\text{fast}})}} + A \times \, (1 - e^{{( - {\text t}/}{\tau\,{\text{fast}})} )} \times (1 - e^{{( - {\text t}/}{\tau\, {\text{slow-attach}})}} ) \times e^{{( - {\text t}/}{\tau\, {\text{slow-detach}})}} } \right], $$

where Force (0), e^(−t/τfast)^, 1 − e^(−t/τfast)^, *A*, 1 − e^(−t/τslow−attach)^, and e^(−t/τslow−detach)^ denote a relative tension level of the maximal Ca^2+^ induced contraction, the number of activated cross-bridges, the number of detached cross-bridges, the rate of re-attachment of the detached cross-bridges, the number of attached slow-cycling (latch-like) state, and the number of detached slow cycling state, respectively (see Fig. 5 and “Discussion” in detail) [17]. The regression analysis was done with a computer program, Kaleida Graph (Synergy Software, Reading, PA, USA) using the Levenberg-Marquart algorithm.

### Statistical analysis

The results are presented as the mean ± S.E.M. Statistical hypotheses on the differences between means were tested using Student's t-test or, analysis of variance (one-way ANOVA; Dunnett’s test). The null hypotheses were rejected when *P* was less than 0.05.

## Results

### Effects of CC-D on the relaxation time course of the skinned taenia cecum and carotid artery muscle preparation

**Fig. 1:**
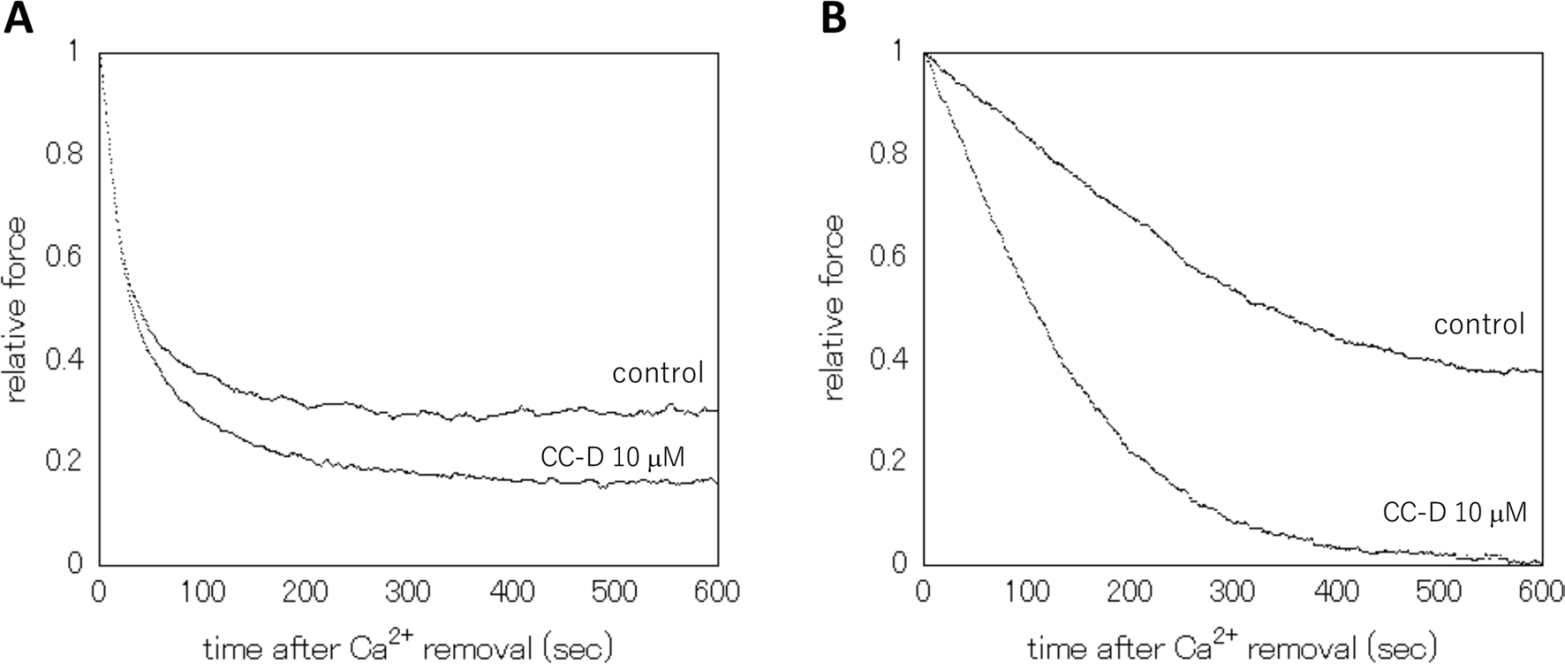
Typical tension traces of relaxation process. Relaxation process after Ca^2+^ removals of β-escin skinned (cell membrane permeabilized) taenia cecum (**A**) and carotid artery (**B**) preparations in the Relaxing solution (with CTP) in the presence or absence of CC-D at 10 μM, 30.0 ± 1.0 ℃. Ordinate: Relative force normalized with the maximal tension level induced by 10^–5.0^ M Ca^2+^ promptly before the Ca^2+^ removal. Abscissa: Time in seconds after the Ca^2+^ removal

When maximally contracting β-escin skinned muscle preparations of guinea pig taenia cecum and carotid artery with 10^–5.0^ M Ca^2+^ were exposed to Relaxing solution (with CTP) for Ca^2+^ removal, the mechanical force fell gradually with an initial fast phase followed by a slow phase, as typically shown in Fig. 1. The time courses of the relaxation processes in both skinned taenia cecum and carotid artery were similar to those in our previous study [10]. The presence of CC-D at 10 μM accelerated relaxations of the taenia cecum and carotid artery, and the relative amount of relaxation was bigger in the carotid artery (about 100% of the maximum tension) than in the taenia cecum (about 80%) (Fig. 1A, B).

**Fig. 2:**
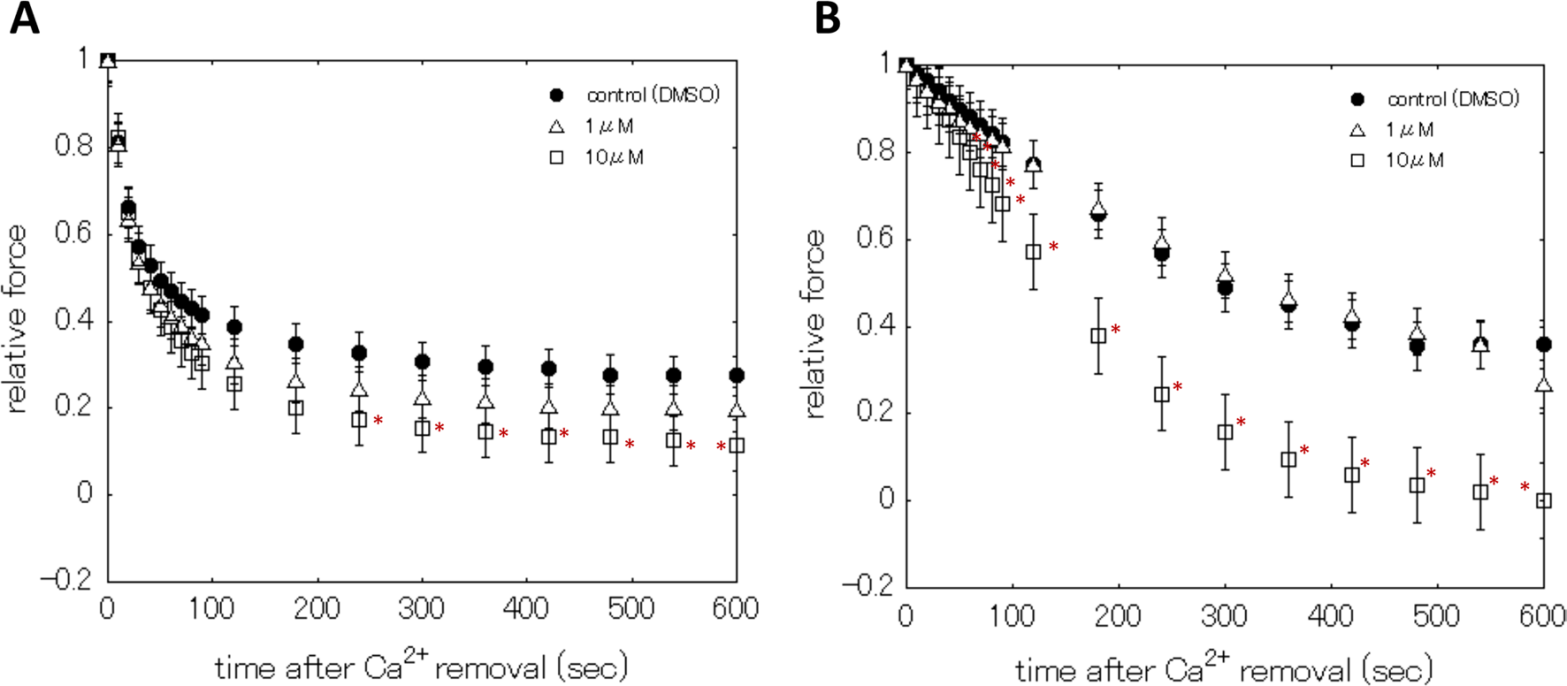
Effects of CC-D on relaxation processes. Statistical representation for the effects of CC-D on the relaxation processes of β-escin skinned taenia cecum (**A**) and carotid artery (**B**). Control (filled circles), CC-D at 1 μM (open triangles), and 10 μM (open squares). Symbols and associated bars are means and ± S.E.M., respectively, tania cecum n = 8, carotid artery n = 8. *Significant difference of the force compared with that of control, where *P* < 0.05

Figure 2 shows the statistical representation of the Ca^2+^ removal-induced relaxation processes of the taenia cecum (Fig. 2A) and carotid artery (Fig. 2B) in the absence and presence of CC-D. The presence of CC-D in the Relaxing solution(with CTP) significantly augmented the relaxation process of the taenia cecum, at time ≧ 240 s for 10 μM. In the case of carotid artery, CC-D at 10 μM significantly elicited the augmentation of relaxation process ≧ 50 s.

### Effects of CC-D on the relaxation process in the rigor condition

**Fig. 3:**
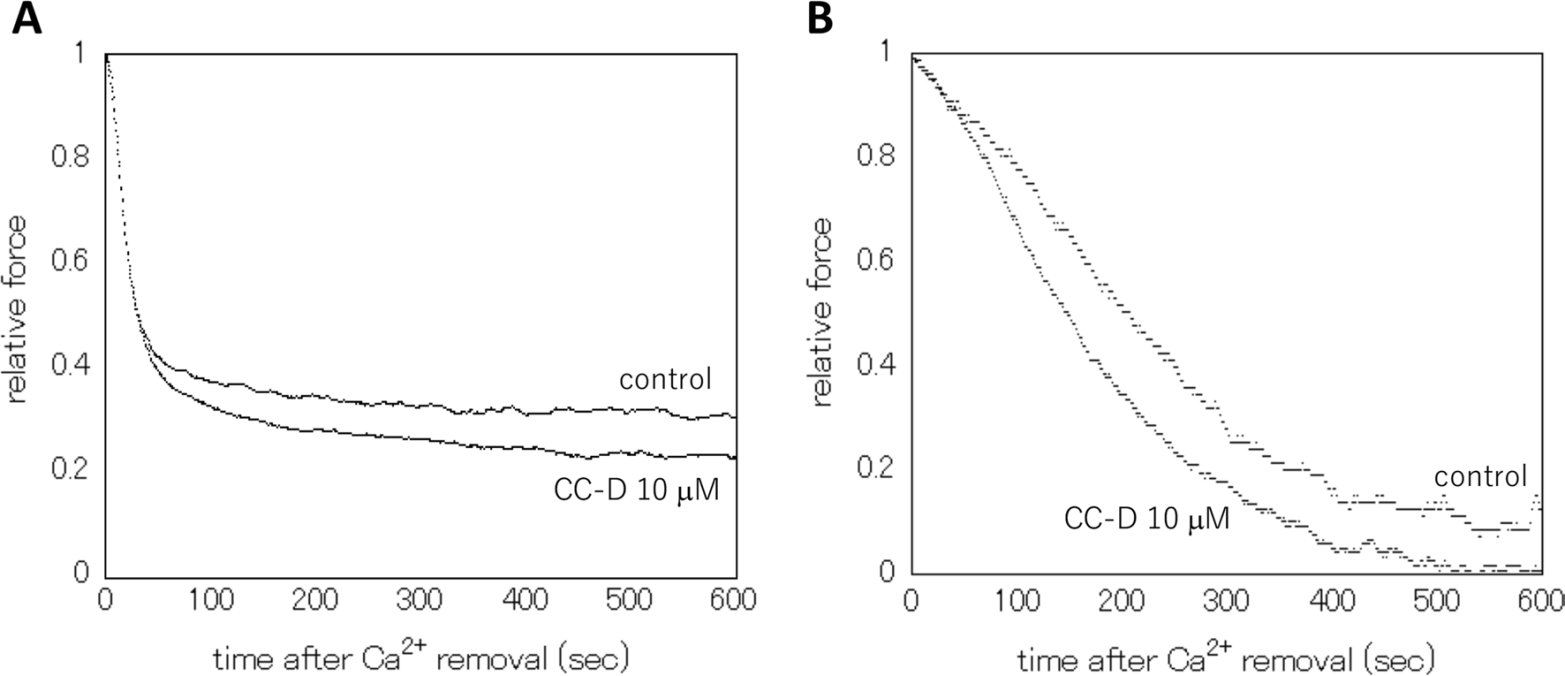
Typical tension traces of relaxation process in the rigor condition. Relaxation process after Ca^2+^ removals of β-escin skinned (cell membrane permeabilized) taenia cecum (**A**) and carotid artery (**B**) preparations in the absence of nucleotide (Rigor condition) in the presence or absence of CC-D at 10 μM, 30.0 ± 1.0 ℃. Ordinate: Relative force normalized with the maximal tension level induced by 10^–5.0^ M Ca^2+^ promptly before the Ca^2+^ removal. Abscissa: Time in seconds after the Ca^2+^ removal

**Fig. 4:**
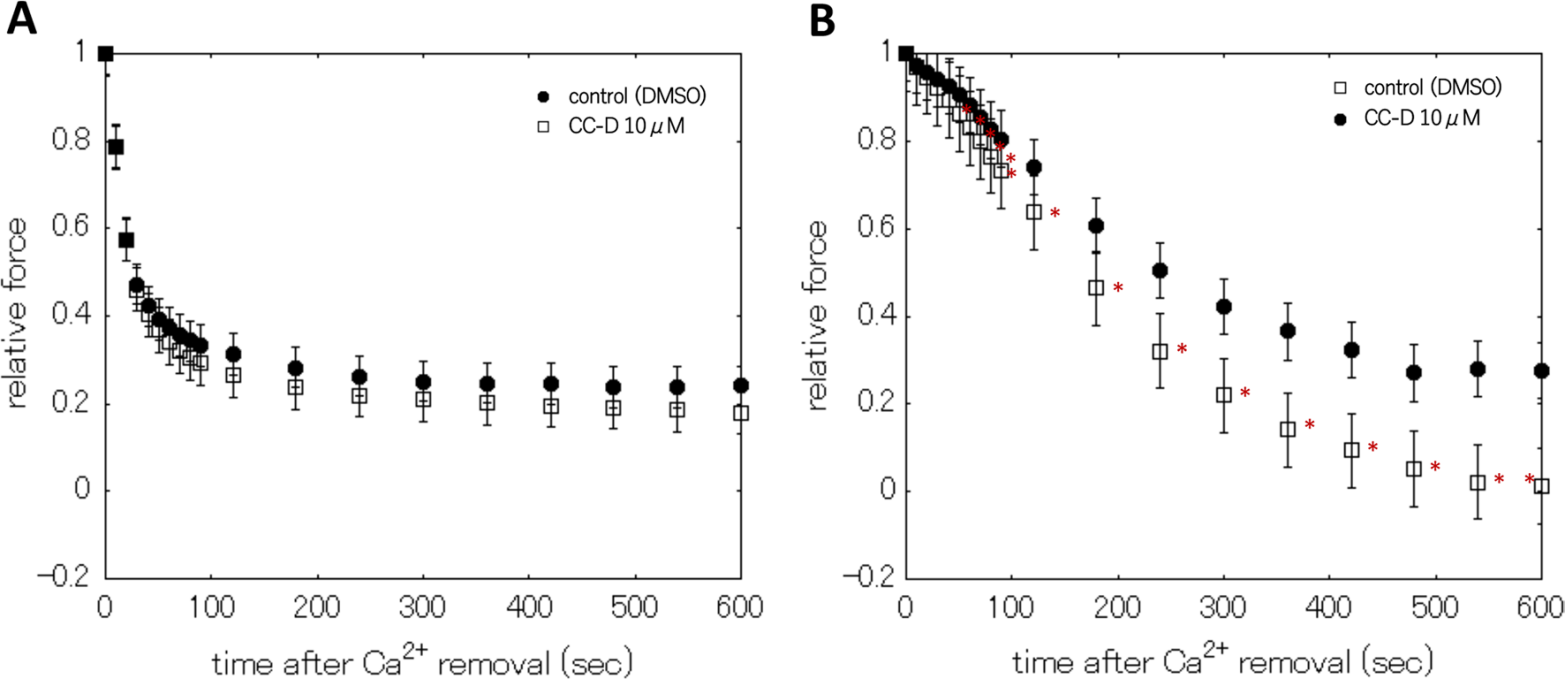
Effects of CC-D on relaxation process in the rigor condition. Effects of CC-D on the relaxation processes of β-escin skinned taenia cecum (**A**) and carotid artery (**B**) in the absence of nucleotide (Rigor condition). Control (filled circles), and CC-D at 10 μM (open squares). Symbols and associated bars are means and ± S.E.M., respectively, n = 8. *Significant difference of the force compared with that of control, where P < 0.05

Figure 3 shows the effects of CC-D on the relaxation process in the absence of nucleotide in Rigor solution (without ATP or CTP). It felled gradually with an initial fast phase followed by a slow phase, similar to normal CTP-contained conditions. In the rigor conditions, CC-D also accelerated the relaxation process of the carotid artery, at time ≧ 40 s at 10 μM (Fig. 4B). On the other hand, in the taenia cecum, the relaxation process was not significantly affected. (Fig. 4A).

### Data fitting analysis

**Fig. 5:**
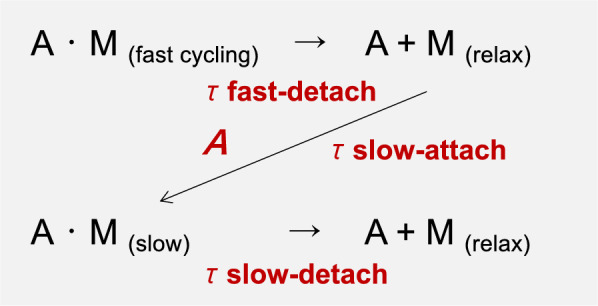
Relaxation process of smooth muscle. The fast cross-bridges (AM fast cycling) activated by Ca^2+^ dissociate once (A + M) with time constant (τ fast-detach) by lowering Ca^2+^ concentration. Then the dissociated myosin binds to actin, and forms slow cycling bridges (AM slow) with time constant (τ slow-attach), and then finally detaches (A + M) with time constant (τ slow-detach). The rate of re-attachment of the detached cross-bridges denotes *A*

Relaxation processes of fast and slow phases in skinned preparations of taenia cecum and carotid artery were analyzed by fitting the Eq. (1), considering with parameters of three constants for fast detaching (τfast-detach), slow attaching (τslow-attach) and slow detaching cross-bridges (τslow-detach), and rate of reattachment of once detached cross-bridges (*A*). The Eq. (1) was based on a kinetic model [17] in which a number of detached myosin from active cross-bridge induced by MLC dephosphorylation re-attaches to actin, then slowly detached from actin again as shown in Fig. 5.

In the control condition with CTP, the τslow-attach (4.0 s) and τslow-detach (1,056.4 s) of carotid artery were about 0.18 and 0.55 times that of taenia cecum, respectively (Table 2). The presence of CC-D at 10 μM with CTP significantly reduced τslow-detach to 0.4 of control in both taenia cecum and carotid artery, which represented the acceleration of detachment process in the slow (latch-like) bridges cycling (Table 2). The presence of CC-D in Relaxing solution (with CTP) elicited a significant reduction in *A* value by 0.6 and 0.33 of control in the taenia cecum and carotid artery, respectively (Table 2). This indicates the reattachment rate of once detached cross-bridges decreased by CC-D treatment.

**Table 2:** Kinetic parameters of the relaxation processes in the presence or absence of CC-D

	Taenia cecum			Carotid artery		
	control	1 μM	10 μM	control	1 μM	10 μM
τfast-detach(sec)	35.3 ± 3.8	41.1 ± 4.4	42.7 ± 2.6	139.3 ± 11.9	156.9 ± 23.9	155.4 ± 10.9
τslow-attach(sec)	21.7 ± 5.1	27.4 ± 4.4	23.3 ± 4.4	4.0 ± 1.2	24.3 ± 9.7	5.4 ± 5.3
τslow-detach(sec)	1,920.6 ± 367.1	1,560.3 ± 369.6	796.9 ± 109.5*	1,056.4 ± 238.3	891.6 ± 166.4	435.2 ± 72.8*
*A*	0.38 ± 0.07	0.29 ± .05	0.23 ± 0.06*	0.67 ± 0.06	0.69 ± 0.08	0.24 ± 0.16*

Kinetic parameters of the relaxation processes of the skinned taenia cecum and carotid artery of the guinea pig in Relaxing solution (with CTP) in the presence or absence of CC-D. Parameters of τfast-detach, τslow-attach, τslow-detach and *A* represent the rate constants of fast detaching, slowly attaching and slow detaching cross-bridge, and the rate of re-attachment of detached crossbridge, respectively. Values represent mean ± S.E.M. (taenia cecum n = 8, carotid artery n = 8) *P < 0.05

Table 3 shows the effects of CC-D at 10 μM on kinetic parameters for relaxation of skinned smooth muscles in Rigor solution (without ATP or CTP). The τrigor-attach (4.6 s) and τrigor-detach (884.6 s) of carotid artery showed similar parameters to the control condition with CTP and were not significantly different. In the taenia cecum and carotid artery exposed to Rigor solution (without ATP or CTP), CC-D elicited marginal and insignificant effects on all parameters tested.

**Table 3:** Effect of CC-D in the absence of nucleotide in Rigor solution

	Taenia cecum		Carotid artery	
	control	10 μM CC-D	control	10 μM CC-D
τfast-detach(sec)	31.2 ± 5.4	26.0 ± 1.6	159.5 ± 12.1	160.5 ± 9.6
τrigor-attach(sec)	45.8 ± 27.6	7.8 ± 3.2*^1^	4.6 ± 1.3	7.8 ± 2.6
τrigor-detach(sec)	1923.2 ± 373.6	1768.1 ± 367.8	884.6 ± 259.1	232.9 ± 53.2*^2^
*A*	0.32 ± 0.07	0.28 ± 0.04	0.59 ± 0.08	0.49 ± 0.11

Kinetic parameters of the relaxation processes of the skinned taenia cecum and carotid artery of the guinea pig in Rigor solution in the presence or absence of CC-D. In addition to parameters listed on Table 2, τrigor-attach and τrigor-detach represent time constants of attaching and detaching rigor-bridge in the rigor conditions, respectively. Values represent mean ± S.E.M. (taenia cecum n = 8, carotid artery n = 8). *^1^P = 0.218. *^2^P = 0.059

## Discussion

The present study shows that, CC-D significantly accelerated the relaxation process by Ca^2+^ removal after Ca^2+^ -induced active tension development in β-escin skinned taenia cecum and carotid artery smooth muscle preparations from guinea pig. CC-D acts specifically on actin to inhibit actin polymerization and growth of actin filaments [18], through its binding to the barbed end of actin filament, and inhibits the binding and dissociation of subunits at the barbed end. Although actin filaments are formed by the helical polymerization of actin monomers, the state of the nucleotide is different at both ends of the actin filament. ATP binds to the barbed end and actin is easily polymerized, and most of the actin filaments changes to ADP by ATP hydrolysis [18]. Also, CC-D binds to actin monomers and dimers, and its binding to dimer accelerates hydrolysis ATP more rapidly [23]. Therefore, CC-D inhibits actin filament growth by capping the barbed end by accelerating ATP hydrolysis cycle to dissociate actin bonds. Regarding the relationship between CC-D and smooth muscle, Saito et al. reported that CC-D inhibits smooth muscle contractions without any effect on the Ca^2+^-dependent MLC phosphorylation, since CC-D may affect neither Ca^2+^ channel activity nor the membrane signaling transductions [13]. In this study, we measured the relaxation process using a Relaxing solution (with CTP) to avoid the effect of CC-D on phosphorylation/dephosphorylation process, since CTP is a substrate for myosin ATPase, but not for other kinases [21, 22]. Thus, as well as in the case of CC-D effects of intact smooth muscle contraction [13], CC-D seems to accelerate relaxation process irrespective of any reaction of phosphorylation/dephosphorylation. Although CTP attenuates actin stability compared to ATP [24], CC-D significantly accelerated relaxation process both in the tania cecum and carotid artery in the presence of ATP (Additional file 1: Fig. S1). Therefore, CC-D effects on relaxation of skinned smooth muscle seem to be not due to nucleoside triphosphate dependent actin stability.

In the present study, CC-D accelerated relaxation process of the skinned smooth muscle around 10 μM. In vitro condition, CC-D has a high affinity for the barbed end of the actin filament even at concentrations as low as 0.2 μM. Also, relatively lower concentrations of CC-D inhibit membrane ruffling and filament growth [18]. On the other hand, higher concentrations (2–20 μM) of CC-D are required to remove stress fibers [18]. Removal of stress fibers means that CC-D binds to actin monomers, which requires large amounts of CC-D. Considering the binding of CC-D not only to filaments but also to free actin monomers, high concentrations would be required [18]. In fact, CC-D at 10 μM is necessary to inhibit intact smooth muscle contraction [13].

It is known that the relaxation process due to the decrease in intracellular Ca^2+^ concentration after smooth muscle contraction is much slower than the MLC dephosphorylation process, and this slower relaxation process has been thought to be due to the latch formation and slow dissociation of dephosphorylated myosin [14]. Previously, based on the kinetic analysis of the relaxation process induced by Ca^2+^ removal in smooth muscle cells, we proposed that, different from “latch bridge theory” in which some MLC phosphorylated cross-bridge directly transfers “latch bridge” after MLC dephosphorylation [14], once MLC dephosphorylated by decrease in intracellular Ca^2+^ concentration induces cross-bridge dissociation, then some dephosphorylated myosin heads newly bind to actin and make slow “latch-like” bridge [17] (see Fig. 5). The regression analysis of the present study indicates that acceleration of relaxation process by CC-D is due to increase in detachment rate of slow cycling (latch-like) bridges, but not to alteration in detachment of fast cycling cross-bridges both in skinned taenia cecum and carotid artery (Table 2). CC-D did not affect fast cross-bridge detachment (τfast-detach) which is the only process dependent of MLC dephosphorylation, indicating that CC-D directly acts the actin structure rather than effecting MLC dephosphorylation. In addition, CC-D also decreases the rate of re-attachment (*A* in Table 2), that indicates suppression of slow cycling (latch-like) bridges formation. Therefore, CC-D seems to directly disrupts actin filament organization or its length, resulting in modulation of actin filament structure that prevents myosin binding.

On the other hand, in regression analysis, the presence of CC-D at 10 μM in Rigor solution (without ATP or CTP) did not have significant effects on the relaxation process both in the skinned taenia cecum and carotid artery (Table 3). The rigor condition is a state in which the myosin head is tightly bound to actin after MgADP dissociation, and the mechanism is different from the relaxation process associated with ATP-induced binding and hydrolysis cycle [25]. Therefore, in the rigor condition without nucleoside triphosphate, actin is tightly bound to myosin, suggesting that CC-D has little effect on slow cycling (latch-like) compared with the effects in the presence of nucleoside triphosphate. However, in the carotid artery, relaxation process was accelerated by CC-D application even in the rigor condition (Fig. 4B). Since CC-D has the effect of disrupting the structure of actin filaments [18], myosin is less likely to bind to the disrupted actin, resulting in accelerated relaxation. There is a difference in the rate of relaxation between the β-escin skinned taenia cecum and the carotid artery, with the taenia cecum having a faster force decay than the carotid artery [10]. In addition, in carotid artery, a tonic smooth muscle, have a higher affinity for myosin MgADP than phasic smooth muscles [26], which may promote larger number of rigor-bridge might be formed compared with that in “phasic” taenia cecum. Therefore, it is considered disruption of actin filament by CC-D may accelerates relaxation process even in the rigor condition only in carotid artery.

## Conclusion

CC-D, an agent directly affects actin structure, weakened actin-myosin binding and promoted relaxation of skinned smooth muscle. Latrunculin B, another inhibitor of actin polymerization, also accelerated skinned smooth muscle relaxation (Additional file 2: Fig. S2). Actin regulation is controlled by variety of mechanisms. The acceleration of depolymerization by CC-D may have secondary effects on actin-tropomyosin linked regulation as well as the inhibition of actin-myosin binding. Further studies are necessary to determine how CC-D affects tropomyosin and actin-tropomyosin binding proteins.

### Supplementary Information

**Additional file 1: Figure S1.** Effect of CC-D on the relaxation process in Relaxing solution (with ATP). Relaxation processes of β-escin skinned taenia cecum (A) and carotid artery (B) in Relaxing solution (with ATP) in the presence or absence of CC-D at 10 μM. Control (filled circles), 10 μM CC-D (open squares). In the taenia cecum, CC-D at 10 μM significantly elicited the augmentation of relaxation process ≧420 s. In the carotid artery, CC-D at 10 μM significantly accelerated the relaxation process for 180, 240, 300, 360, and 420 s. Symbols and associated bars are means and ± S.E.M., respectively, taenia cecum n = 2, and carotid artery n = 3 *Significant difference of the force compared with that of control, where P < 0.05.

**Additional file 2: Figure S2.** Effects of Latrunculin B on relaxation processes. Statistical representation for the effects of Latrunculin B on the relaxation processes of β-escin skinned taenia cecum (A) and carotid artery (B). Control (filled circles), and Latrunculin B at 1 μM (open triangles), and 10 μM (open squares). In the carotid artery, latrunculin B significantly accelerated the relaxation process at 10 μM for 120, 180, 240, and 300 s. Symbols and associated bars are means and ± S.E.M., respectively, taenia cecum n = 8, carotid artery n = 8. *Significant difference of the force compared with that of control, where P < 0.05.

## Data Availability

The datasets used and/or analyzed during the current study are available from the corresponding author on reasonable request.
