# Supplementary figures and images for: Effects of cytochalasin D on relaxation process of skinned taenia cecum and carotid artery from guinea pig
Source: J Physiol Sci. 2024 Apr 10;74:24. doi: 10.1186/s12576-024-00918-3 (PMC11007923; doi:10.1186/s12576-024-00918-3)

**A**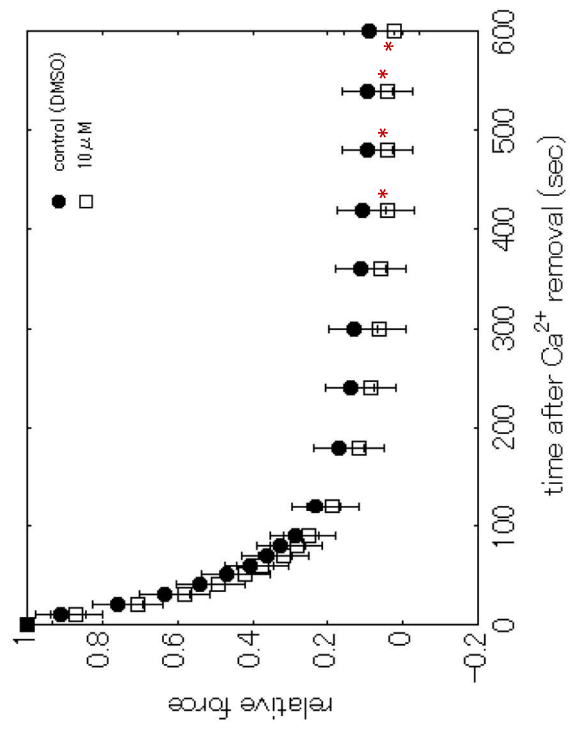**B**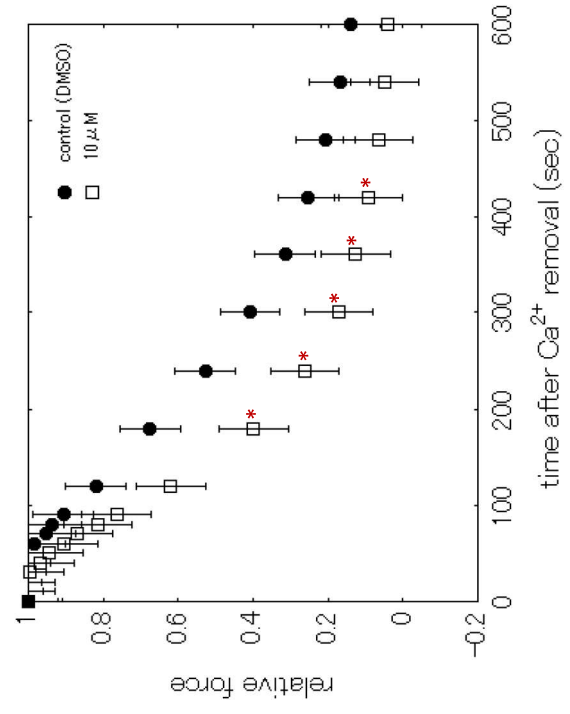

Supplement: Supplementary file 1 — Additional file 1: Figure S1. Effect of CC-D on the relaxation process in Relaxing solution (with ATP). Relaxation processes of β-escin skinned taenia cecum (A) and carotid artery (B) in Relaxing solution (with ATP) in the presence or absence of CC-D at 10 μM. Control (filled circles), 10 μM CC-D (open squares). In the taenia cecum, CC-D at 10 μM significantly elicited the augmentation of relaxation process ≧420 s. In the carotid artery, CC-D at 10 μM significantly accelerated the relaxation process for 180, 240, 300, 360, and 420 s. Symbols and associated bars are means and ± S.E.M., respectively, taenia cecum n = 2, and carotid artery n = 3 *Significant difference of the force compared with that of control, where P < 0.05. [file 12576_2024_918_MOESM1_ESM.pdf]

**A**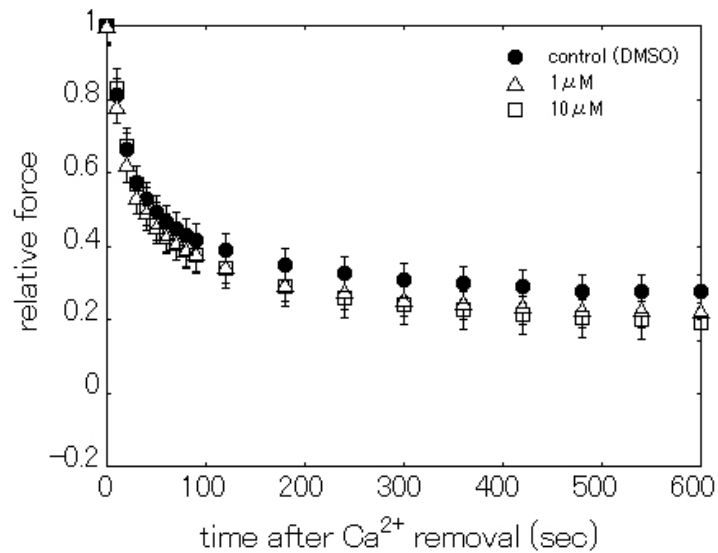**B**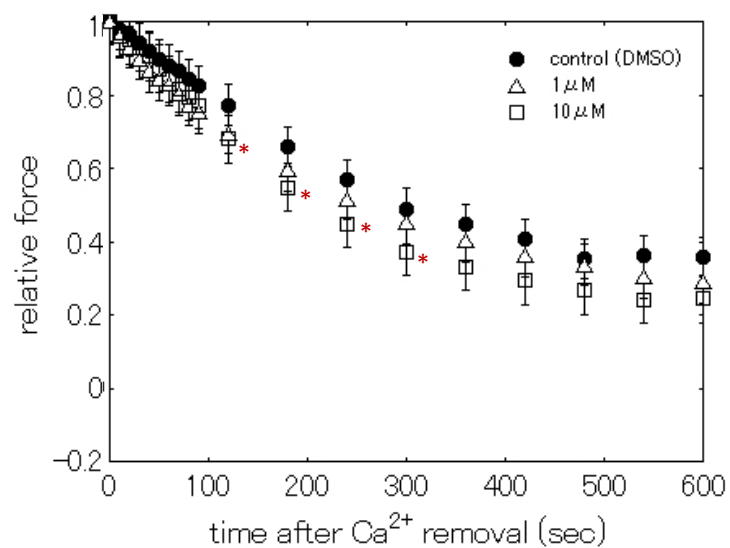

Supplement: Supplementary file 2 — Additional file 2: Figure S2. Effects of Latrunculin B on relaxation processes. Statistical representation for the effects of Latrunculin B on the relaxation processes of β-escin skinned taenia cecum (A) and carotid artery (B). Control (filled circles), and Latrunculin B at 1 μM (open triangles), and 10 μM (open squares). In the carotid artery, latrunculin B significantly accelerated the relaxation process at 10 μM for 120, 180, 240, and 300 s. Symbols and associated bars are means and ± S.E.M., respectively, taenia cecum n = 8, carotid artery n = 8. *Significant difference of the force compared with that of control, where P < 0.05. [file 12576_2024_918_MOESM2_ESM.pdf]
